# Ninjinyoeito improves social behavior disorder in neuropeptide Y deficient zebrafish

**DOI:** 10.3389/fphar.2022.905711

**Published:** 2022-08-12

**Authors:** Momoko Kawabe, Takumi Nishida, Chihoko Horita, Asami Ikeda, Ryuji Takahashi, Akio Inui, Kazuhiro Shiozaki

**Affiliations:** ^1^ Course of Biological Science and Technology, The United Graduate School of Agricultural Sciences, Kagoshima University, Kagoshima, Japan; ^2^ Department of Food Life Sciences, Faculty of Fisheries, Kagoshima University, Kagoshima, Japan; ^3^ Kampo Research Laboratories, Kracie Pharma Ltd., Toyama, Japan; ^4^ Pharmacological Department of Herbal Medicine, Graduate School of Medical and Dental Sciences, Kagoshima University, Kagoshima, Japan

**Keywords:** sociability, zebrafish, herbal medicine, Ninjinyoeito, neuropeptide Y

## Abstract

Sociability is an essential component of the linkage structure in human and other vertebrate communication. Low sociability is defined as a poor social approach, including social withdrawal and apathy, and is implicated in a variety of psychiatric disorders. Ninjinyoeito (NYT), a traditional Japanese herbal medicine, has been used in the medical field. This study aimed to determine the effect of NYT on low sociality in NPY-KO zebrafish. NPY-KO zebrafish were fed a 3% NYT-supplemented diet for 4 days and subjected to behavioral tests. In the mirror test, NPY-KO zebrafish fed a control diet showed avoidance behavior toward their mirror counterparts. In contrast, the treatment of NPY-KO zebrafish with NYT significantly increased their interaction with their counterparts in the mirror. In addition, a 3-chambers test was conducted to confirm the effect of NYT on the low sociality of NPY-KO zebrafish. NPY-KO zebrafish fed the control diet showed less interaction with fish chambers, while NYT treatment increased the interaction. Phosphorylation of ERK, a marker of neuronal activity, was significantly reduced in the whole brain of NYT-fed NPY-KO zebrafish, compared to the control diet. NYT treatment significantly suppressed hypothalamic-pituitary-adrenal-related genes (*gr*, *pomc*, and *crh*) and sympathetic-adrenal-medullary-related genes (*th1*, *th2*, and *cck*) in NPY-KO zebrafish. NYT administration significantly reduced mRNA levels of *gad1b* compared to the control diet, suggesting the involvement of GABAergic neurons in NYT-induced improvement of low sociability. Furthermore, the expression of CREB was suppressed when NPY-KO zebrafish were fed NYT. Next, we attempted to identify the effective herb responsible for the NYT-induced improvement of low sociability. NPY-KO zebrafish were fed an experimental diet containing the target herb for 4 days, and its effect on sociability was evaluated using the 3-chambers test. Results showed that Cinnamon Bark and Polygala Root treatments significantly increased time spent in the fish tank area compared to the control diet, while the other 10 herbs did not. We confirmed that these two herbs suppressed the activity of HPA-, SAM-, and GABAergic neurons, as well as NYT-treated zebrafish, accompanied by downregulation of CREB signaling. This study suggests the potential use of NYT as a drug for sociability disorders.

## 1 Introduction

Social behavior is defined as social communication, such as sociability and mating, between individuals of similar species (Geng and Peterson, 2019). Among social behaviors, sociability is an essential factor in the social linkage structure in humans and other vertebrates. In general, low sociability is defined as an inferior social approach, such as social withdrawal or apathy (Radke et al., 2014; Wilson and Koenig, 2014). Recently, the hypothalamic-pituitary-adrenal (HPA) axis has been shown to regulate sociability (Qiao et al., 2014; Vasconcelos et al., 2019; Hughes et al., 2020). The production and release of corticotropin-releasing hormone (CRH) stimulate the production and release of pro-opiomelanocortin (POMC)-derived adrenocorticotropic hormone (ACTH), which boosts glucocorticoid synthesis in the HPA axis (Skobowiat et al., 2011). High levels of CRH and POMC induce low sociability in mammals (Qiao et al., 2014; Hughes et al., 2020). Sociability plays a role in various psychiatric disorders, with the degree of symptoms differing based on the patients.

To develop drugs to improve low sociability, adequate animal models that show apparent symptoms are required (Geng and Peterson, 2019). Rodents have been used as model animals for social disorders because they are highly sociable and possess many social behavior traits similar to that in humans (Geng and Peterson, 2019). Furthermore, several protocols have been established to assess sociabilities, such as the mirror test and the 3-chambers test (Spencer et al., 2005; Vasconcelos et al., 2019). Recently, animal models other than rodents have also been studied. Zebrafish (*Danio rerio*) is a popular animal model in behavioral neuroscience research owing to its easy maintenance, low cost, and abundant offspring compared to rodents (Rosa et al., 2018). Zebrafish exhibit high sociability and conserve many genes involved in regulating social behavior in humans and rodents (Ariyasiri et al., 2019). In addition, protocols for assessing sociability have been established in zebrafish, such as the mirror test and the 3-chambers test, similar to those used in rodents (Ariyasiri et al., 2019; Shiozaki et al., 2020). Therefore, zebrafish are suitable for drug development and medical screening to improve their sociability. Recently, neuropeptide Y knockout (NPY-KO) zebrafish were established as models exhibiting low social behavior (Shiozaki et al., 2020). NPY has been implicated in regulating emotional behavior, such as social and anxiety behaviors, in humans (Ueda et al., 2021). The zebrafish NPY amino acid sequence is highly similar to human NPY (89%), accompanied by a similar NPY function as humans (Singh et al., 2017; Shiozaki et al., 2020). NPY-KO zebrafish exhibited low social behavior with a reduced mirror approach in the mirror test (Shiozaki et al., 2020).

The present study focused on the use of herbal medicine as a drug to improve sociability. These medicines are composed of various herbs and have synergistic effects on human diseases (Zhou et al., 2016). Recently, herbal medicines have attracted attention in psychiatric disorders (Miyano et al., 2018). Ninjinyoeito (NYT) is a traditional Japanese herbal medicine comprising 12 herbs (Miyano et al., 2018; Kawabe et al., 2021). NYT improves anorexia, cold limbs, fatigue, and anemia (Murata et al., 2018). Several studies have suggested the involvement of the NYT in the HPA axis. NYT improves desperate behavior and memory in mice *via* inhibition of the HPA axis (Murata et al., 2018). NYT prevents neuropathic pain with chronic contractile injury in mice *via* the HPA axis (Takemoto et al., 2021). Although the HPA axis is involved in sociability regulation, the effect of NYT on increasing sociability has not been thoroughly studied. Here, to evaluate the impact of NYT on low sociability, this study employed NPY-KO zebrafish fed a NYT-supplemented diet, followed by an analysis of the alteration of social behavior. Furthermore, we tried to identify the active herb components in NYT and clarify NYT’s mechanism. We have previously reported anxiolytic effects of NYT on acutely stressed NPY-KO zebrafish (Kawabe et al., 2021). On the other hand, the present study evaluated the effects of NYT on the sociability of NPY-KO zebrafish in the absence of stress.

## 2 Materials and methods

### 2.1 Zebrafish

NPY-KO zebrafish were generated by genome editing of CRISPR/Cas9 using the RIKEN WT (RW) with 11 nucleotide deletion of the first exon, which induces mistranslation of NPY polypeptides due to frameshift (Shiozaki et al., 2020). In this study, the wild-type RW strain was used as the WT. The zebrafish were housed in a 2-L water tank with a 14/10 h light/dark photoperiod cycle. Live brine shrimp and a commercial diet (Otohime B2, Marubeni Nissin Feed Ltd., Tokyo, Japan) were provided twice daily for zebrafish. This study used adult zebrafish aged 6–12 months. The Kagoshima University Committee approved all protocols used in this study of Animal Experiments. This study was performed following the relevant guidelines and regulations.

### 2.2 Administration of ninjinyoeito and its herbal medicine in zebrafish

NYT (lot no. 16033006) and each herbal medicine in NYT, including Rehmannia Root (lot no. T160526), Japanese Angelica Root (lot no. T160621), Atractylodes Rhizome (lot no. T160549), Poria Sclerotium (lot no. T160681), Ginseng (lot no. T160623), Cinnamon Bark (lot no. T160583), Polygala Root (lot no. T170006), Peony Root (lot no. T160615), Citrus Unshiu Peel (lot no. T160582), Astragalus Root (lot no. T160598), Glycyrrhiza (lot no. T160536), and Schisandra Fruit (lot no. T160523), were supplied as a freeze-dried powder of boiling water extract by Kampo Research Laboratories (Kracie Pharma, Ltd., Toyama, Japan) (Murata et al., 2018). All herbs information was listed in Table 1. Each plant material was identified by external morphology and authenticated by marker compounds of plant specimens according to the method of Japanese Pharmacopeia and our company’s standard. For the quality check of NYT, NYT extract was mixed and shaken with 50% methanol and the supernatant was subjected to high-performance liquid chromatography (HPLC) analysis. The three-dimensional HPLC profile of NYT was obtained using a Shimazu Nexera X3 system with an SPD-M40 detector with scanning for a range of 190–450 nm and a reversed-phase column [ACQUITY UPLC® BEH C18 1.7 μm (2.1 mm × 100 mm, 1.7 μm), Column temperature: 40°C]. The column was equipped with solvent A (0.1% formic acid in water) and solvent B (0.1% formic acid in methanol), and the ratio of solvent A was increased by A/B 90/10-90/10-5/95 (0-10-45 min), with a flow rate at 0.3 ml/min.

**TABLE 1:** Formulas of NYT.

Component herbs	Family name	Species name	Weight (g)^a^
Rehmanniae Radix (Rehmannia root)	Plantaginaceae	*Rehmannia glutinosa* (Gaertn.) DC.	4
Angelicae acutilobae Radix (Japanese Angelica root)	Apiaceae	*Angelica acutiloba* (Siebold and Zucc.) Kitag.	4
Atractylodis Rhizoma (Atractylodes rhizome)	Compositae	*Atractylodes japonica* Koidz. ex. Kitam.	4
Poria (Poria sclerotium)	Polyporaceae	*Wolfiporia cocos* Ryvarden et Gilbertson	4
Ginseng Radix (Ginseng)	Araliaceae	*Panax ginseng* C.A. Mey.	3
Cinnamomi Cortex (Cinnamon bark)	Lauraceae	*Cinnamomum cassia* (L.) J. Presl	2.5
Polygalae Radix (Polygala root)	Polygalaceae	*Polygala tenuifolia* Wild.	2
Paeoniae Radix (Peony root)	Paeoniaceae	*Paeonia lactiflora* Pall.	2
Citri unshiu Pericarpium (Citrus unshiu peel)	Rutaceae	*Citrus unshiu* Markowicz	2
Astragali Radix (Astragalus root)	Polygalaceae	*Astragalus mongholicus* Bunge	1.5
Glycyrrhizae Radix (Glycyrrhiza)	Fabaceae	*Glycyrrhiza uralensis* Fisch. ex DC.	1
Schisandrae Fructus (Schisandra fruit)	Schisandraceae	*Schisandra chinensis* (Turcz.) Baill.	1

a Amount of herbs for the preparation of 6.7 g NYT extract.

NYT-containing diet was prepared as follows: NYT was mixed with the powdered commercial diet at a concentration of 3% or 0.3%, and then freeze-dried and re-formed into pellet (0.6–1.0 mm). Each herbal medicine was added as an equivalent of 3% or 0.3% of NYT with a commercial diet. Extraction efficiencies herbs were 0.673, 0.375, 0.41, 0.029, 0.309, 0.095, 0.219, 0.354, 0.274, 0.345, 0.294, and 0.393 for Rehmannia Root, Japanese Angelica Root, Atractylodes Rhizome, Poria Sclerotium, Ginseng, Cinnamon Bark, Polygala Root, Peony Root, Citrus Unshiu Peel, Astragalus Root, Glycyrrhiza, and Schisandra Fruit, respectively. The control diet was prepared as described above without NYT or herbal medicines. The diets were stored at −20°C during the feeding experiment.

The feeding experiment was carried out in 2-L tanks with water at 28°C. The zebrafish (0.8 g average weight) were fed a commercial diet until the feeding experiment initiation. The fish were divided into 2-L tanks. They were fed the experimental diet twice daily for 4 days. Food intake by the zebrafish was recorded daily.

### 2.3 Evaluation of social behaviors

#### 2.3.1 Mirror test

**FIGURE 1:**
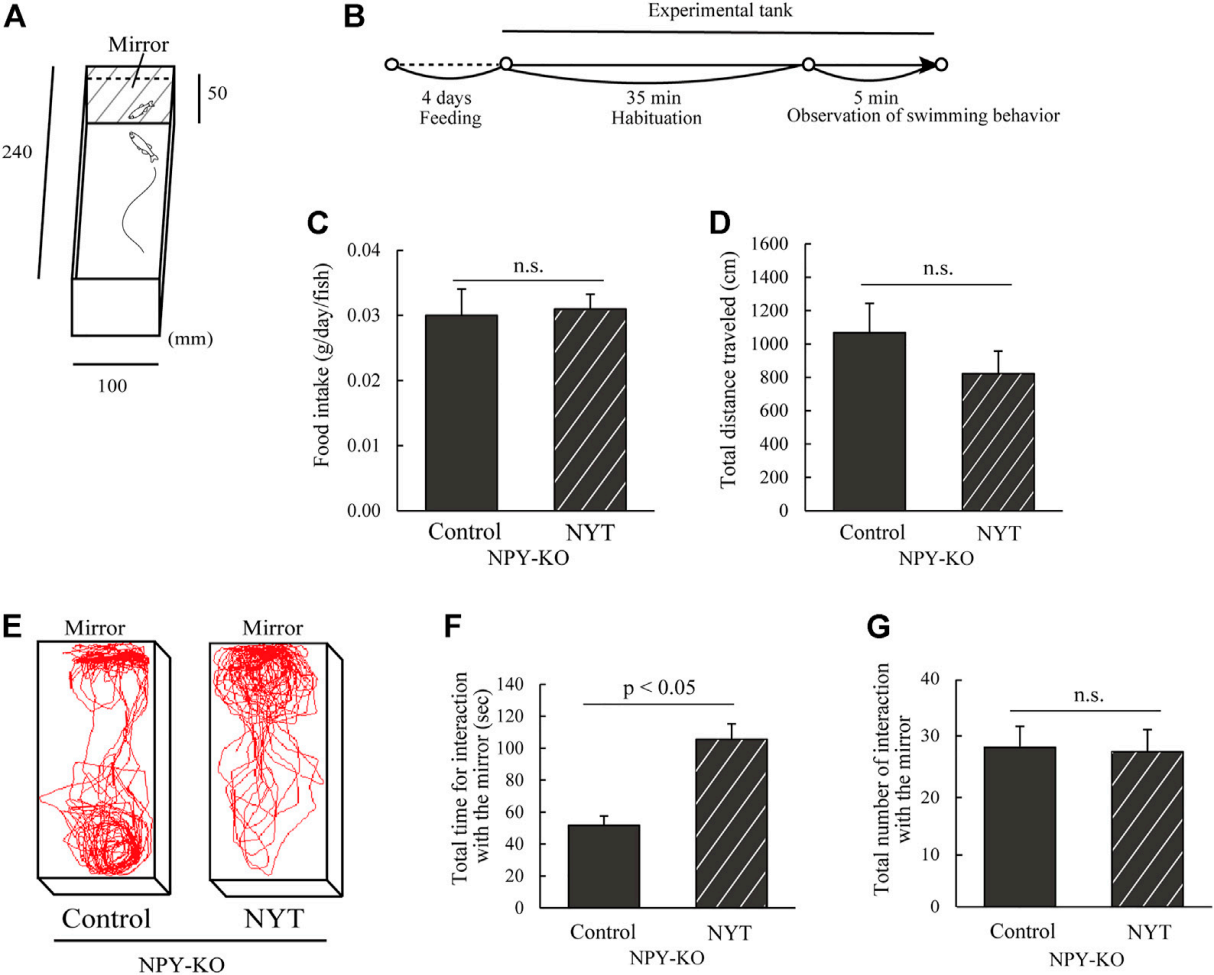
Effect of NYT on social behavior on mirror test in NPY-KO zebrafish. NPY-KO zebrafish were fed a control or NYT-diet twice daily for 4 days. Their social behaviors, such as interaction with the mirror and swimming distance, were estimated using a mirror test. **(A)** Mirror test apparatus. **(B)** Experimental scheme for behavioral evaluation. **(C)** Daily food intake. **(D)** Total distance traveled. **(E)** Tracking of control (left) and NYT-fed zebrafish (right) swimming behavior. **(F)** Total time for interaction with the mirror. **(G)** Total number of interactions with mirror. *n* = 8. Results are shown as mean ± standard error of the mean. n.s., not significant.

According to a previous study, the mirror test was conducted with slight modifications (Shiozaki et al., 2020). The tank was 5 cm high, 10 cm wide, and 24 cm long, and a mirror was placed on one side (Figure 1A). Eight zebrafish were used for each treatment. A fish was placed in the center of a test aquarium, with a white board covering the front of the mirror. After acclimation for 35 min, the board was removed, and the fish behavior was recorded for 5 min using a digital video camera (HDR-CX430, SONY, Tokyo, Japan) (Figure 1B). In this study, swimming parallel to the opponent in the mirror was defined as social behavior (Shiozaki et al., 2020). When the fish approached the mirror, the angle was vertical. After approaching, the fish exhibited parallel swimming with the opponent in the mirror (defined as interaction) or turned back without interaction. The total number of interactions, the amount of time of the interaction, the total distance traveled, and swimming tracking were analyzed using Move-tr/2D software (Library, Tokyo, Japan).

#### 2.3.2 Aggression test

**FIGURE 2:**
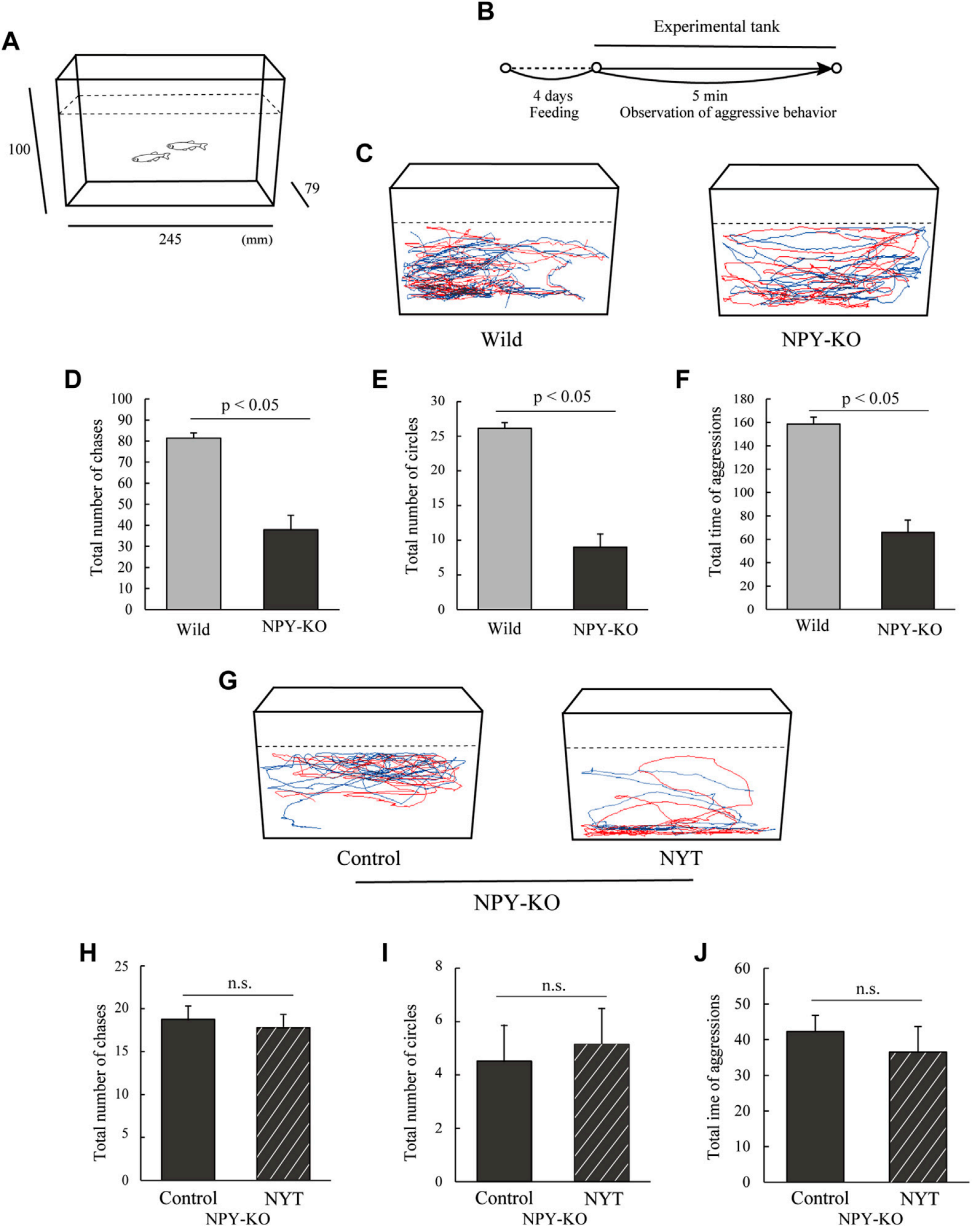
Effect of NYT on aggressive behavior in NPY-KO zebrafish. Wild and NPY-KO zebrafish were fed either control or NYT twice a day for 4 days. Two unfamiliar male zebrafish were set in an aquarium, and their aggressive behavior was analyzed. **(A)** Test apparatus. **(B)** Experimental scheme for behavioral evaluation. **(C)** Tracking of wild (left) and NPY-KO zebrafish (right) swimming behavior. **(D)** Total number of chases in the WT and NPY-KO zebrafish. **(E)** Total number of circles in the WT and NPY-KO zebrafish. Wild, *n* = 6; NPY-KO, *n* = 7. **(F)** Total time of aggression in wild and NPY-KO zebrafish. **(G)** Tracking of control (left) and NYT-fed (right) swimming behavior in NPY-KO zebrafish. **(H)** Total number of chases in control and NYT-fed NPY-KO zebrafish. **(I)** Total number of circles in control and NYT-fed NPY-KO zebrafish. **(J)** Total aggression time in control and NYT-fed NPY-KO zebrafish. *n* = 6. n.s., not significant. Results are shown as mean ± standard error of the mean.

As described previously, aggressive behavior was conducted with slight modifications (Ikeda et al., 2021). The tank was 10 cm in height, 7.9 cm long and 24.5 cm wide (Figure 2A). Seven pairs of male zebrafish were used for each treatment. Two male zebrafish were transferred into the tank, and their behaviors were recorded for 5 min using a digital camera (Figure 2B). Aggressive behaviors were defined as chasing towards the opponent and circling. The total number of chases, the number of circles, and the total time of aggressive behaviors were recorded. Swimming tracking was obtained from the first 1 min of the behavioral data using Move-tr/2D software.

#### 2.3.3 Three-chambers test

**FIGURE 3:**
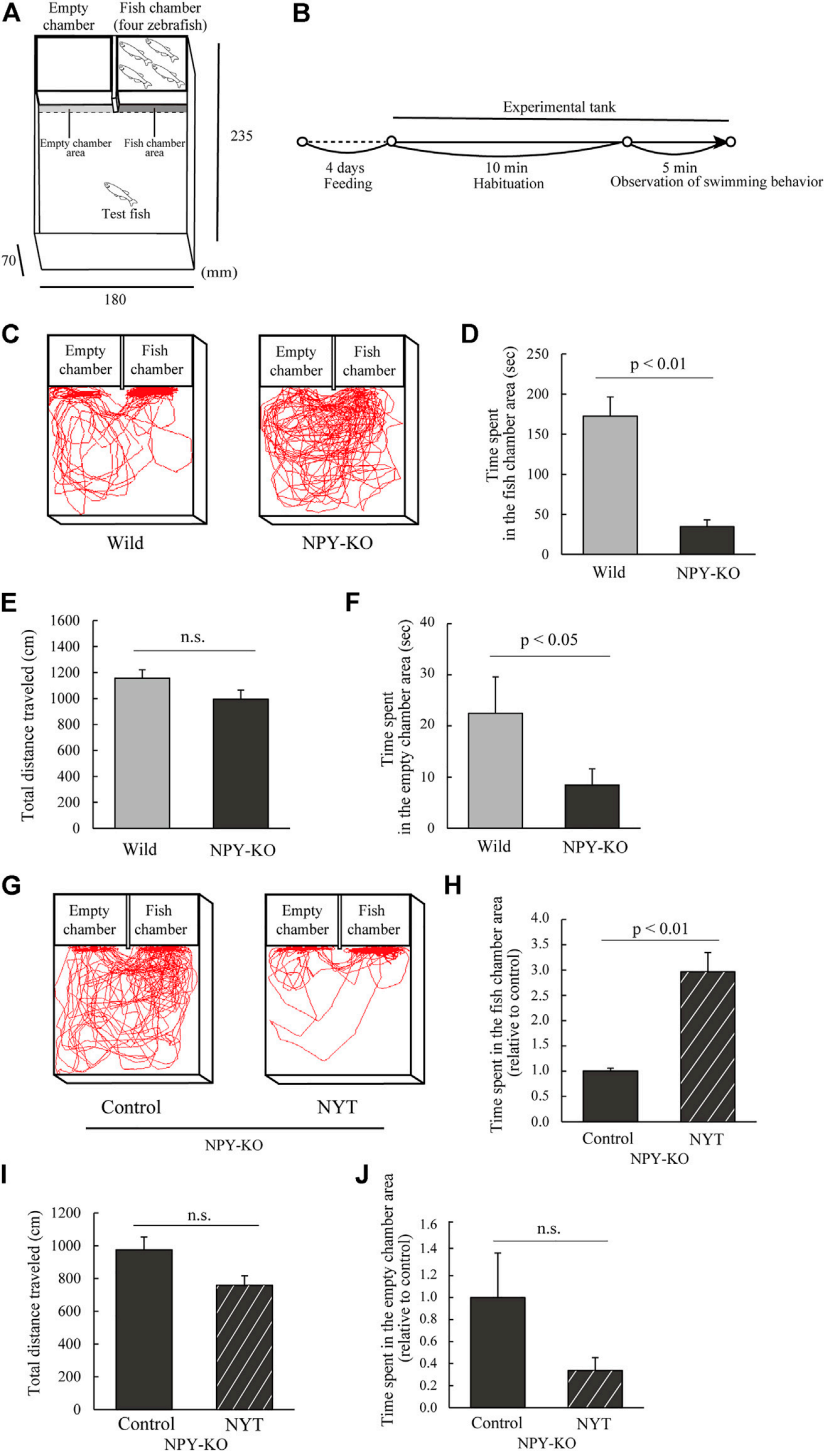
Effect of NYT on sociability in NPY-KO zebrafish. Wild and NPY-KO zebrafish were fed either control or NYT twice a day for 4 days. Sociability was analyzed using the 3-chambers test. **(A)** Test apparatus. **(B)** Experimental scheme for behavioral evaluation. **(C)** Tracking of wild (left) and NPY-KO zebrafish (right) swimming behavior. **(D)** Time spent in the fish chamber area in wild and NPY-KO zebrafish. **(E)** Total distance traveled in the WT and NPY-KO zebrafish. **(F)** Time spent in the empty chamber area of wild and NPY-KO zebrafish. Wild, *n* = 9; NPY-KO, *n* = 8. **(G)** Tracking of control (left) and NYT-fed (right) NPY-KO zebrafish swimming behavior. **(H)** Time spent in the fish chamber area of the control and NYT-fed NPY-KO zebrafish. **(I)** Total distance traveled by control and NYT-fed NPY-KO zebrafish. **(J)** Time spent in the empty chamber area in control and NYT-fed NPY-KO zebrafish. *n* = 8. Results are shown as mean ± standard error of the mean. n.s., not significant.

As described elsewhere, the experiment was conducted with slight modifications (Ikeda et al., 2021). Two small transparent chambers (9.1 cm × 5.3 cm × 5.5 cm) were placed on one side of the experimental tank (23.5 cm × 18 cm × 7 cm) (Figure 3A). Eight to nine zebrafish were used as test fish for each treatment. These small chambers are called empty chamber and fish chamber, respectively. Then, two male and two female zebrafish were placed in the fish chamber, whereas the other remained empty. A test fish was placed in the center of the tank and acclimated for 10 min after being transferred to a test aquarium, where the front of the chamber was covered with a white board. The board was then removed, and the fish behavior was recorded for 5 min using a digital video camera (Figure 3B). The data for the time spent in the fish and empty chamber area (the 0.5 cm area in the fish and empty chamber area), the total distance traveled, and swimming tracking were analyzed using Move-tr/2D software.

### 2.4 Real-time PCR

The mRNA expression levels of genes were analyzed using zebrafish brain cDNA with a StepOne Real-Time System (Thermo Fisher Scientific, MA, United States). Five fish from each treatment were used for the analysis. The zebrafish were sacrificed using 0.1% tricaine. The fish brains were removed and immediately frozen at −80°C until RNA extraction. The total RNA was extracted from fish brains using Sepasol-RNA Ι Super G (Nacalai Tesque, Kyoto, Japan). cDNA was synthesized with ReverTra Ace qPCR RT Master Mix with qPCR Remover (TOYOBO, Osaka, Japan). Real-time PCR was performed using KOD SYBR qPCR Mix (TOYOBO) with specific primers for glucocorticoid receptor (*gr*), proopiomelanocortin (*pomc*), corticotropin-releasing hormone (*crh*), isotocin (homolog of human oxytocin, *ist*), arginine vasotocin (homolog of human vasopressin, *avt*), orexin (*orx*), tyrosine hydroxylase 1 (*th1*), *th2*, cholecystokinin (*cck*), dopamine transporter (*dat*), glutamic acid decarboxylase 1b (*gad1b*), *gad2*, tryptophan hydroxylase 1a (*tph1a*), *tph1b*, *tph2*, brain-derived neurotrophic factor (*bdnf*), and cAMP response element-binding protein 1 (*creb1*) genes. The primers used are listed in Supplementary Table S1. The PCR was carried out at the following conditions: 2 min at 98°C, 10-s denaturation step at 98°C, 10-s annealing step at 60°C, and 30-s extension step at 68°C for 40 cycles. The expression level of the *actb* mRNA was used to compensate for the quality and quantity of each sample.

### 2.5 Immunoblotting

Zebrafish brains were lysed in 50 mM 4-(2-hydroxyethyl)-1-piperazineethanesulfonic acid (pH 7.4), 150 mM NaCl, 1% NP-40, 2 mM ethylenediaminetetraacetic acid, 10 mg/ml leupeptin, 10 mM sodium fluoride, 2 mM sodium orthovanadate, 0.25% sodium deoxycholate, and 2 mM phenylmethylsulfonyl fluoride. Five fish from each treatment were used for the analysis. Lysates were separated using a 10% acrylamide gel and transferred to a polyvinylidene difluoride (PVDF) membrane. The membrane was blocked with 1% bovine serum albumin (BSA) in PBS containing 0.1% Tween 20 (PBST) and incubated with anti-phospho ERK and anti-ERK (polyclonal; 1/1,000 dilution; Cell Signaling Technology, MA, United States), anti-β-actin (clone 2D4H5; 1/1,000 diluted; Proteintech, IL, United States), and anti-phospho CREB and anti-CREB (monoclonal, 10E9, D-12, respectively. 1/500 dilution; Santa Cruz Biotechnology, TX, United States). After incubation with a horseradish peroxidase-conjugated secondary antibody, target protein bands were detected using EzWestLumi plus chemiluminescence reagent (ATTO, Tokyo, Japan) using ChemiDoc Touch Plus (Bio-Rad, CA, United States). Densitometric analysis was performed using Image Lab Touch software (Bio-Rad).

### 2.6 Data analysis

Results are presented as mean ± standard error of the mean. All values were compared using Student’s *t*-test. Three or more groups were compared using one-way analysis of variance (ANOVA) followed by Tukey’s multiple comparison test.

## 3 Results

### 3.1 Improvement of sociability in Ninjinyoeito-fed zebrafish

#### 3.1.1 Mirror test

Supplementary Figure S1 shows a 3D-HPLC profile of NYT along with a chemical analysis. Chemical makers, such as paeoniflorin, hesperidin, and glycyrrhizic acid, were used for quality control. This study used NPY-KO zebrafish that demonstrate low social behavior in the mirror test to evaluate the influence of NYT on low social behavior (Shiozaki et al., 2020). The experimental scheme used for the mirror test is shown in Figure 1B. During the feeding period, NYT did not affect the food intake in the NPY-KO zebrafish, which was similar to the control group (Figure 1C). No other adverse events were observed in NYT-fed zebrafish, like NYT-fed mice (Takaku et al., 2017). NYT did not affect the total distance traveled in the NPY-KO zebrafish in the test tank, similar to that for the control diet (Figure 1D). Compared to the control diet, NYT-fed NPY-KO zebrafish spent more time interacting with the mirror (2.0-fold increase in NYT vs. control; *p* < 0.05; Figures 1E,F), but the overall number of interactions with the mirror did not change (Figure 1G). This result indicates that NYT enhanced the interaction with the mirror in the NPY-KO zebrafish.

#### 3.1.2 Aggression test

In general, the mirror test results reflect the behavior of sociability or aggression (Ikeda et al., 2021). Therefore, to clarify the effects of NYT on aggressive behavior, the aggressive behavior was analyzed in NYT-fed NPY-KO zebrafish (Figures 2A,B). First, the aggressive behavior of NPY-KO zebrafish was evaluated by comparison with the WT. NPY-KO zebrafish exhibited decreased total number of chases (53.4% decrease to WT; *p* < 0.05, Figures 2C,D) and circling (66.0% decrease to WT; *p* < 0.05, Figures 2C,E) compared to WT. The total time of chases and circles in NPY-KO zebrafish decreased compared with WT zebrafish (58.5% decrease to WT; *p* < 0.05, Figure 2F). These results indicated low aggressive behavior in NPY-KO zebrafish. Next, we evaluated the effect of NYT on low aggressive behavior in NPY-KO zebrafish. Compared to the control diet, NYT did not affect the total number of chases, circling, and the total time of aggression in NPY-KO zebrafish (Figures 2G–J). Taken together with the results of Figures 1, 2, the increased approach toward the opponent in the mirror in the NYT-fed group may not be due to aggression.

#### 3.1.3 Three-chambers test

To clarify the effect of NYT on sociability, NYT-fed NPY-KO zebrafish were applied for the 3-chambers test to evaluate the interaction of zebrafish with unfamiliar zebrafish (Figure 3A) (Ikeda et al., 2021). In this test, sociability was assessed by the interaction with four zebrafish. First, the interaction of NPY-KO zebrafish was compared with that of the WT. NPY-KO zebrafish exhibited decreased time spent in the fish chamber area compared with the WT zebrafish (79.8% decrease to WT; *p* < 0.01; Figures 3C,D), while the total distance travelled by both groups did not change (Figure 3E). The time spent in the empty chamber area in NPY-KO zebrafish also decreased compared with that in wild zebrafish (72.4% decrease to WT; *p* < 0.05; Figures 3C,F). These results indicate low sociability in NPY-KO zebrafish.

Next, we assessed the effects of NYT on NPY-KO zebrafish behavior in the 3-chambers test. NPY-KO zebrafish that were fed NYT showed increased time spent in the fish chamber compared to that fed the control diet (2.9-fold increase; *p* < 0.01; Figures 3G,H) without any changes in the total distance traveled (Figure 3I) and time spent in the empty chamber (Figure 3J). These results suggest that NYT improves the interaction behaviors in NPY-KO zebrafish.

### 3.2 Alteration of social behavior-related molecules in Ninjinyoeito-fed zebrafish

**FIGURE 4:**
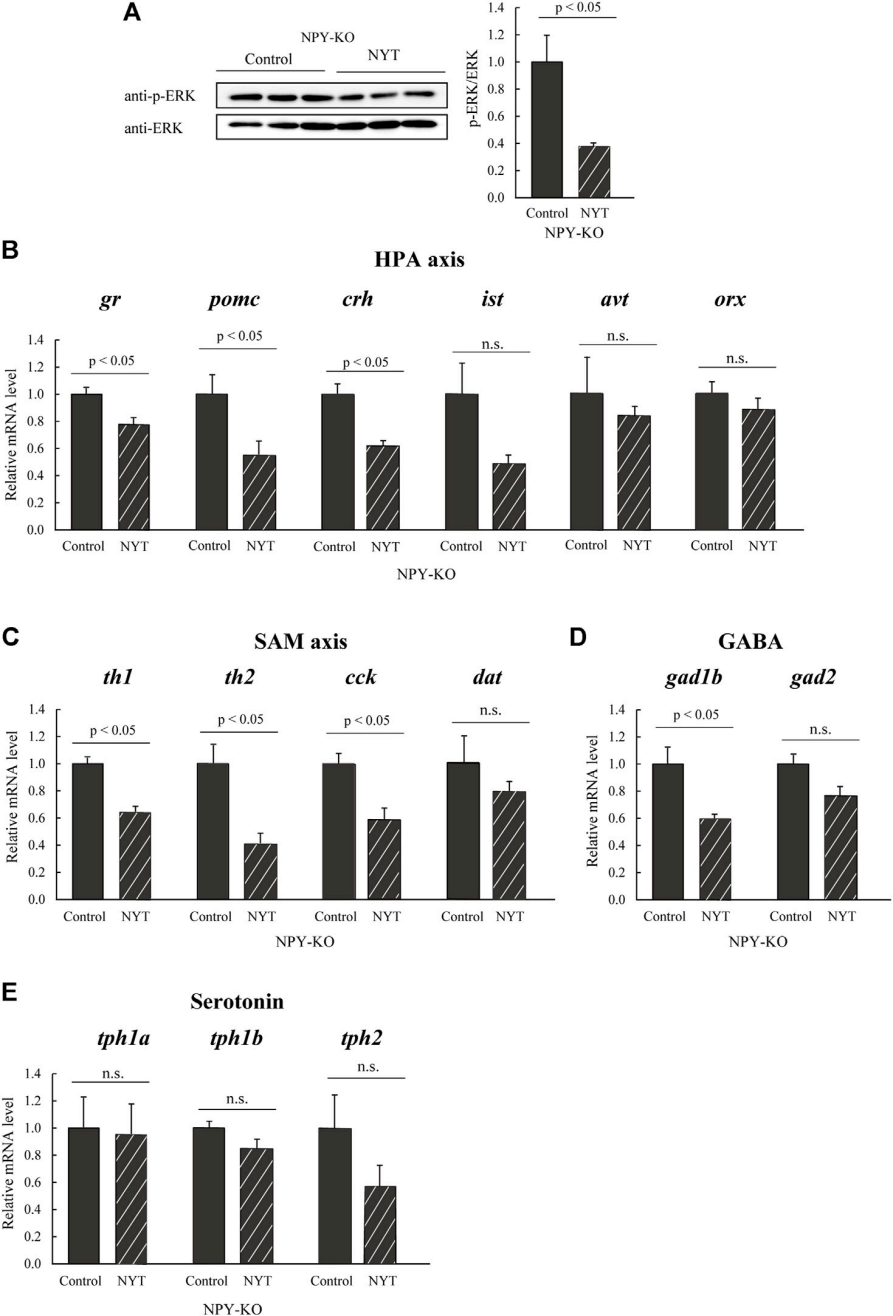
Effect of NYT on social behavior-related molecules in the NPY-KO zebrafish. The protein levels of **(A)** p-ERK were analyzed by immunoblotting with whole-brain lysate of the control—and NYT-fed NPY-KO zebrafish. The loading control used was **(A)** total ERK. *n* = 4. The mRNA levels of **(B)** HPA axis, **(C)** SAM axis, **(D)** GABA, and **(E)** serotonin-related genes were estimated by real-time PCR using whole brains of the control—and NYT-fed NPY-KO zebrafish. **(B)** HPA axis-related genes (*gr*, *pomc*, *crh*, *ist*, *avt*, and *orx*). **(C)** SAM axis-related genes (*th1*, *th2*, *cck*, and *dat*). **(D)** GABA-related genes (*gad1b* and *gad2*). **(E)** Serotonin-related genes (*tph1a*, *tph1b*, and *tph2*). The gene expression level of *actb* was used as an internal reference. Each gene expression level was relative to that in the control-fed NPY-KO zebrafish. *n* = 5. Results are shown as mean ± standard error of the mean. n.s., not significant.

The expression levels of genes and proteins linked to social behavior and neural activation were evaluated using real-time PCR and immunoblotting analysis to understand better the molecular mechanisms underlying the improved social interaction behavior of NYT seen in NPY-KO zebrafish. ERK phosphorylation, a marker of neuronal activation marker (Lu et al., 2019), was significantly decreased in NYT-fed zebrafish compared to that of control (60.0% decrease compared to control; *p* < 0.05; Figure 4A). The expression levels of HPA axis-related genes (*gr*, *pomc*, *crh*, *ist*, and *avt*), the sympathetic-adrenal-medullary (SAM) axis-related genes (*th1*, *th2*, *cck*, and *dat*), HPA and SAM axis related genes (*orx*), GABA-related genes (*gad1b* and *gad2*), and serotonin-related genes (*tph1a*, *tph1b*, and *tph2*) were estimated by real-time PCR. Among the HPA-related genes, NYT treatment resulted in significant suppression of *gr*, *pomc*, and *crh* expression levels compared with the that fed with the control diet in NPY-KO (21.3%, 43.7%, and 31.3% decrease to control, respectively; *p* < 0.05), whereas the mRNA levels of *ist* and *avt* were not altered (Figure 4B). NYT treatment also significantly decreased the expression of *th1*, *th2*, and *cck*, but not of *dat* and *orx*, compared with those that were fed the control diet (37.3%, 62.3%, and 48.3% decrease to control, respectively; *p* < 0.05; Figure 4B), NYT treatment significantly decreased *gad1b* (40.2% decrease; *p* < 0.05, Figure 4D), but not *gad2*, compared to the control group (Figure 4D). NYT did not affect the mRNA levels of *tph1a*, *tph1b*, and *tph2* (Figure 4E). These results indicated that NYT treatment attenuated neuronal function in the HPA axis, SAM axis, and GABA neurons.

**FIGURE 5:**
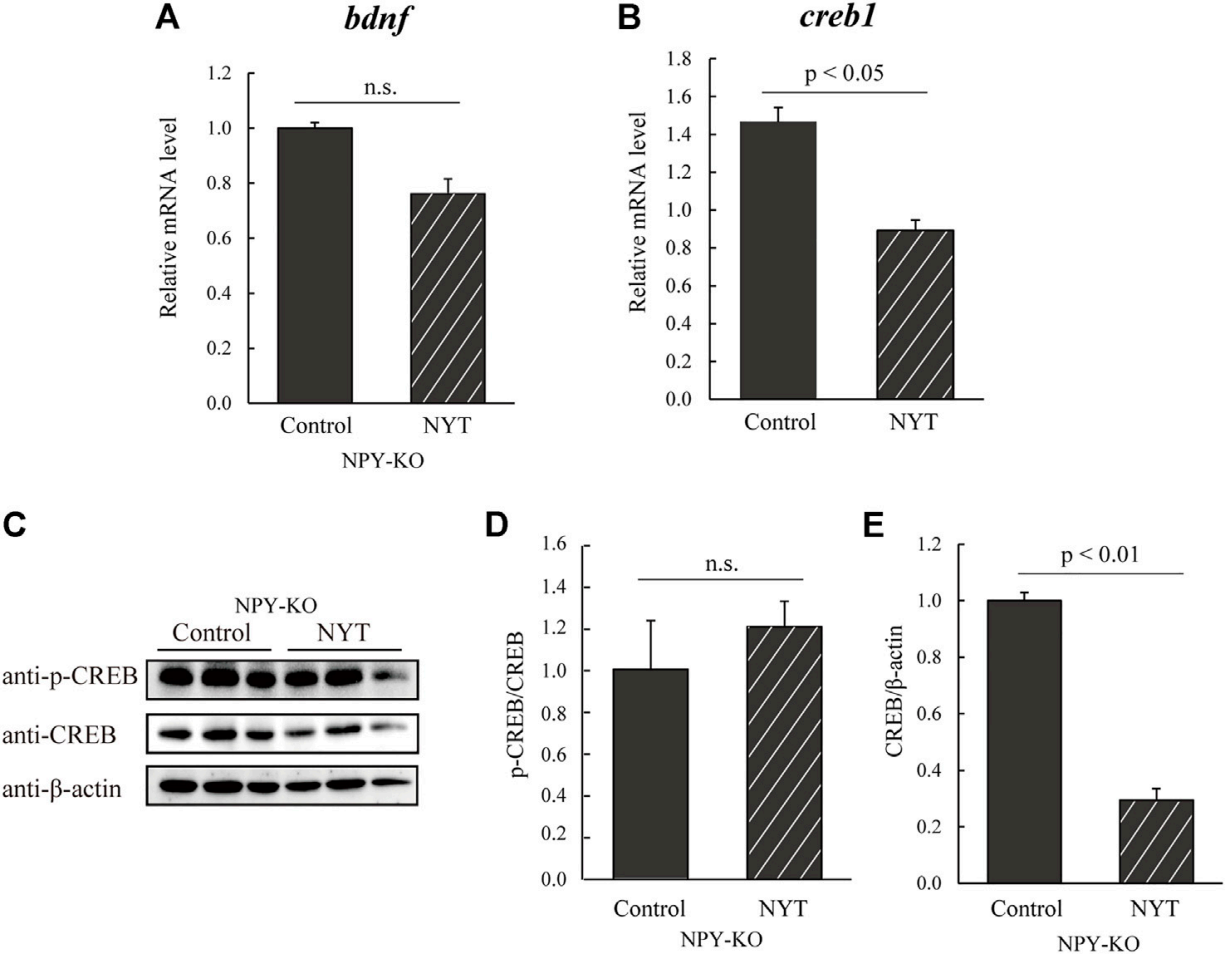
Effect of NYT on BDNF/CREB in NPY-KO zebrafish. The mRNA levels of *bdnf* and *creb1* were estimated by real-time PCR using the whole brain of the control—and NYT-fed NPY-KO zebrafish. **(A)** *bdnf*. **(B)** *creb1*. The gene expression level of *actb* was used as an internal reference. Each gene expression level was relative to that in the control-fed NPY-KO zebrafish. *n* = 5. The protein levels of **(C,D)** p-CREB and **(C,E)** CREB were analyzed using immunoblotting with whole-brain lysates of control—and NYT-fed NPY-KO zebrafish. The loading control used was **(D)** total CREB and **(E)** β-actin. *n* = 4. Results are shown as mean ± standard error of the mean. n.s., not significant.

CREB increases HPA and SAM axis-related gene expression and *bdnf* (Paşca et al., 2011; Tafet and Nemeroff, 2020). In addition, the stimulation of glucocorticoid, dopamine (DA), and noradrenaline (NA) receptors activate CREB (Xu et al., 2006; Matsui and Sugie, 2017; Wang et al., 2018). Here, we estimated the expression levels of *bdnf* and *creb1* using real-time PCR. While *bdnf* mRNA levels in the NYT treatment did not differ from that in the control group (Figure 5A), *creb1* was downregulated in NYT compared to control (39.5% decrease to control; *p* < 0.05; Figure 5B). Furthermore, the phosphorylation of CREB did not differ in the two tested groups (Figures 5C,D), while NYT treatment significantly decreased CREB protein levels (70.5% decrease; *p* < 0.01; Figures 5C,E). These results indicate that NYT treatment attenuated the expression of CREB protein, possibly resulting in the decline of the HPA/SAM axis.

### 3.3 Evaluation of effective herbal medicines in Ninjinyoeito for the improvement of social behavior

**FIGURE 6:**
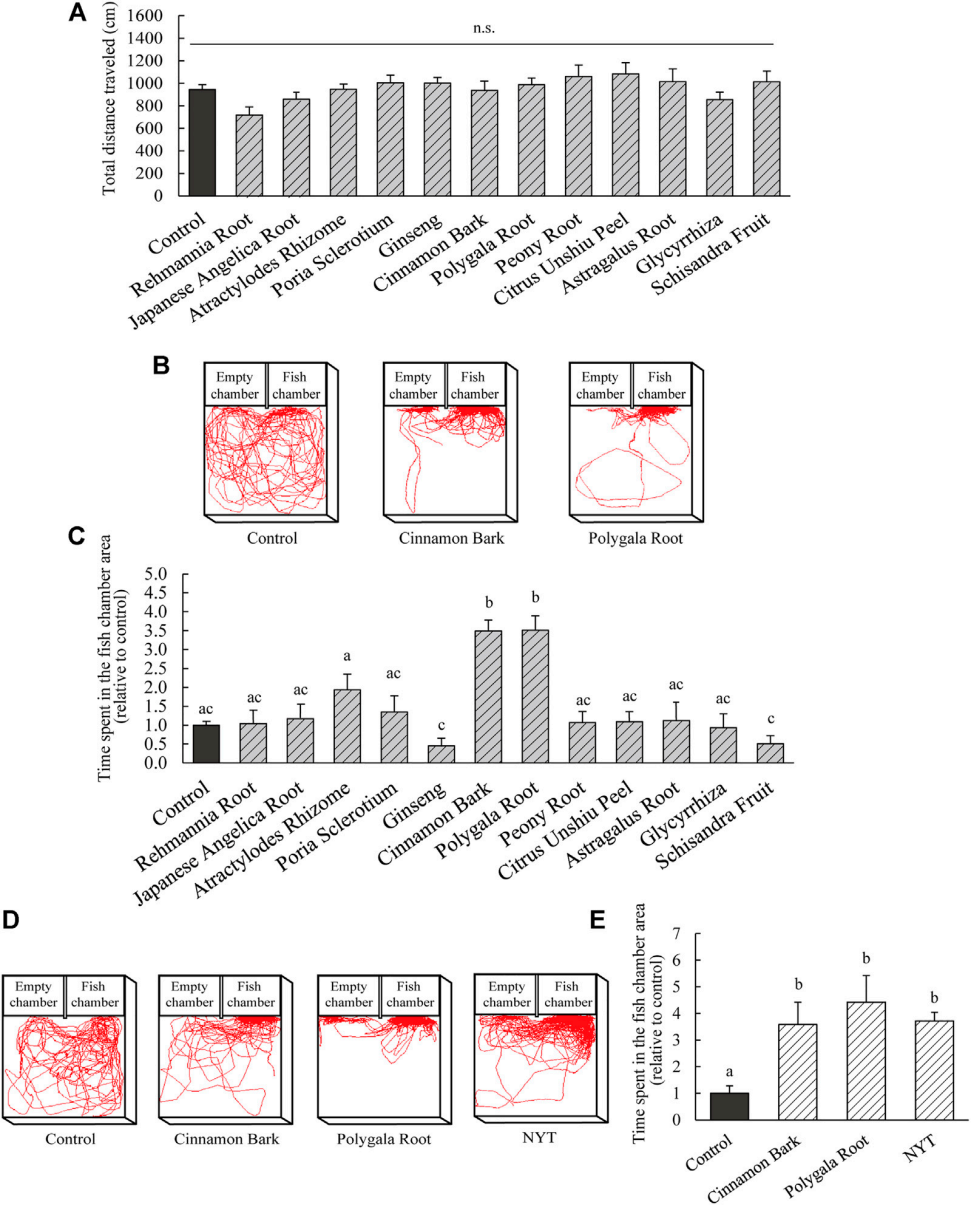
Evaluation of improvement of sociability in herbal medicine originating from NYT. **(A–E)** NPY-KO zebrafish were fed a control and herbal medicine diet twice a day for 4 days. Sociability was analyzed using the 3-chambers test. **(A)** Total distance traveled by control—and herbal medicine-fed NPY-KO zebrafish (equivalent to 3% NYT). **(B)** Tracking of control (left), Cinnamon Bark (middle), and Polygala Root-fed (right) swimming behavior. **(C)**Time spent in the control and herbal medicine-fed NPY-KO zebrafish in the fish chamber area. Control, *n* = 41; herbal medicines*, n* = 8. **(D)** Tracking of control (left), Cinnamon Bark (middle), and Polygala Root fed (middle), NYT (right) swimming behavior (equivalent to 0.3% NYT). **(E)** Time spent in the fish chamber area in control, Cinnamon Bark, Polygala Root, and NYT-fed NPY-KO zebrafish. Control *n* = 8; herbal medicines *n* = 7. Results are shown as mean ± standard error of the mean. There were significant differences between the different signs. n.s., not significant.

The present study revealed an improvement in the sociability of NPY-KO zebrafish by NYT. To identify the effective herb(s) in NYT, an experimental diet containing each herbal medicine (equivalent to 3% of NYT) was fed to NPY-KO zebrafish. Their sociability was analyzed using a 3-chambers test. None of the used herbal medicines affected the food intake in the zebrafish (Supplementary Figure S2A). No other adverse events were observed in the herb-fed fish. The total distance traveled did not differ between the control and all herbal medicine treatment groups (Figure 6A). Cinnamon Bark and Polygala Root treatments increased the time spent in the fish chamber area compared with the control diet (3.4-fold and 3.5-fold increase to control, respectively; *p* < 0.05; F = 14.34, *p* < 0.0001, one-way ANOVA; Figures 6B,C). The time spent in the empty chamber area in the Atractylodes Rhizome treatment increased compared with the control (3.4-fold increase compared to the control; *p* < 0.05; F = 3.09, *p* < 0.001, one-way ANOVA; Supplementary Figure S2B).

To compare the effectiveness of Cinnamon Bark and Polygala Root, the social behavior of NPY-KO zebrafishes fed the herbs were evaluated in the 3-chambers test (0.3% equivalent to NYT). The herbal medicines and NYT did not affect the food intake in NPY-KO zebrafish with a lower concentration of NYT/herbs (0.3%) (Supplementary Figure S2C). Treatment with Cinnamon Bark, Polygala Root, and NYT increased the time spent in the fish chamber area compared to the control diet (3.6-, 4.4-, and 3.7-fold increase, respectively; *p* < 0.005; F = 5.87, *p* < 0.05, one-way ANOVA; Figures 6D,E). Cinnamon Bark, Polygala Root, and NYT were comparable in respect to the time spent in the fish chamber area. The total distance traveled and time spent in the empty chamber area were not affected by Cinnamon Bark, Polygala Root, or NYT (Supplementary Figures S2D,E). These results suggest that the herbs responsible for improving abnormal sociability by using NYT are Cinnamon Bark and Polygala Root.

**FIGURE 7:**
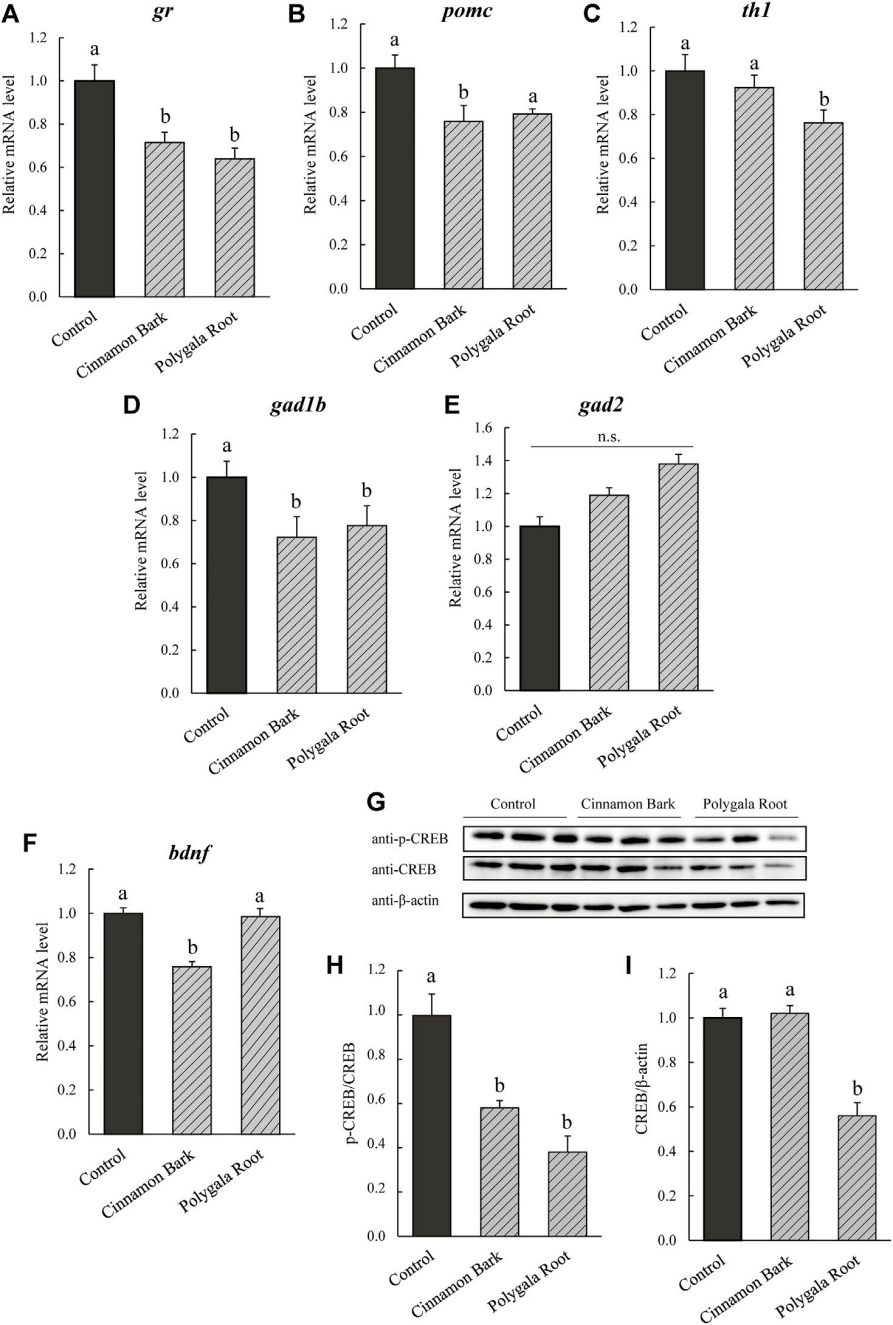
Effect of Cinnamon Bark and Polygala Root on BDNF/CREB in NPY-KO zebrafish. mRNA levels of the **(A,B)** HPA axis, **(C)** SAM axis, **(D,E)** GABA -related gene, and **(F)** *bdnf* were estimated by real-time PCR using whole brains of the control—and Cinnamon Bark -and Polygala Root-fed NPY-KO zebrafish. **(A)** *gr*. **(B)** *pomc*. **(C)** *th1*. **(D)** *gad1d*. **(E)** *gad2*. **(F)** *bdnf*. The gene expression level of *actb* was used as an internal reference. Each gene expression level was relative to that in the control-fed NPY-KO zebrafish. *n* = 5. The protein levels of **(G,H)** p-CREB and **(G,I)** CREB were analyzed by immunoblotting with whole-brain lysate of the control—and herbal medicine-fed NPY-KO zebrafish. A loading control was used as the **(H)** total CREB and **(I)** β-actin. *n* = 8. Results are shown as mean ± standard error of the mean. There were significant differences between the different signs. n.s., not significant.

The expression levels of genes related to the HPA and SAM axes and GABA neurons were estimated in Cinnamon Bark and Polygala Root-fed NPY-KO zebrafish (equivalent to 3% NYT). *gr* was downregulated by Cinnamon Bark and Polygala Root treatment compared with the control diet (28.4% and 35.9%, respectively; *p* < 0.05; F = 12.23, *p* < 0.005, one-way ANOVA; Figure 7A). *pomc* of the HPA axis-related gene was downregulated by Cinnamon Bark compared with the control diet (24.3% decrease compared to the control; *p* < 0.05; F = 6.00, *p* < 0.05, one-way ANOVA; Figure 7B). In addition, *th1* was downregulated by Polygala Root compared with the control diet (23.8% decrease; *p* < 0.05; F = 8.706, *p* < 0.05, one-way ANOVA; Figure 7C). Cinnamon Bark and Polygala Root treatment decreased *gad1b* mRNA level compared with the control diet (25.6% and 19.4% decrease to control; *p* < 0.05; F = 8.192, *p* < 0.001, one-way ANOVA; Figure 7D), *gad2* did not differ between the Cinnamon Bark and Polygala Root treatments (Figure 7E). In addition, the expression levels of *bdnf* decreased in Cinnamon Bark (33.3% decrease; *p* < 0.05; F = 18.54, *p* < 0.001, one-way ANOVA), whereas Polygala Root did not differ from the control diet (Figure 7F). Compared to the control diet, the treatment of NPY-KO zebrafish with Cinnamon Bark and Polygala Root attenuated CREB phosphorylation (43.4% and 61.6% decrease, respectively; *p* < 0.05; F = 17.57, *p* < 0.001, one-way ANOVA; Figures 7G,H). CREB protein levels decreased in Polygala Root treatment compared with the control diet (43.6% decrease; *p* < 0.05; F = 31.36, *p* < 0.0001, one-way ANOVA; Figures 7G–I), while Cinnamon Bark did not differ from the control diet (Figures 7G,I). These results indicate that Cinnamon Bark treatment attenuated the HPA axis and GABA neurons. Whereas Polygala Root treatment inhibited the HPA and SAM axis and GABA neurons, accompanied by the downregulation of CREB signaling.

## 4 Discussion

This study revealed the improvement in sociability by NYT and its mechanism using NPY-KO zebrafish. NYT enhanced the interaction of NPY-KO zebrafish with opponents in the mirror and with unfamiliar zebrafish groups *via* attenuation of the HPA and SAM axis and the GABA neurons. We identified Cinnamon Bark and Polygala Root as herbs responsible for developing sociability by NYT.

Patients with social withdrawal and autism show activation of the HPA axis (Roberts et al., 2009). The high CRH levels decrease the social approach in a 3-chambers test in rodents, and CRH antagonists improve this behavior (Dunn and File, 1987; Vasconcelos et al., 2019). POMC induced by a high ACTH level decreases social approach (Qiao et al., 2014) and social interactions such as grooming and sniffing (Malkesman et al., 2006). The signaling system of the human HPA axis is also conserved in rodents and zebrafish (Piato et al., 2011). GR mutants have higher cortisol levels in zebrafish and show a reduced social approach (Bonan and Norton, 2015). This study found that NYT treatment downregulated the expression of HPA-related genes (*gr*, *crh*, and *pomc*) in NPY-KO zebrafish. In contrast, AVP and OXT are involved in regulating social behavior through the attenuation of the HPA axis (Storm and Tecott, 2005; Rault et al., 2013). In addition, ORX receptor-deficient mice have impaired social behavior, such as the social approach in the 3-chambers test (Abbas et al., 2015; Faesel et al., 2021). However, improvement of abnormal sociability by NYT would be independent of Avt, Ist, and Orx because there was no alteration in the expression of these genes in NYT-treated NPY-KO zebrafish.

Previous studies have reported that the HPA axis is innervated by the SAM axis, serotonin neurons, and GABA neurons (Lanfumey et al., 2008; Kakizawa et al., 2016; Bao and Swaab, 2019). NA neurons activate CRH neurons in the SAM axis (Valentino and Van Bockstaele, 2008; Serova et al., 2019). GABA neurons also activate the HPA and SAM axes (Kakizawa et al., 2016; Atmore et al., 2020). NYT therapy reduced the expression of SAM-related genes (*th1*, *th2*, and *cck*) but not serotonin neuron markers (*tph1a, tph1b,* and *tph2*), implying that NYT treatment suppresses the HPA axis by downregulating the SAM axis and GABA neurons.

CREB is known to be one of the factors regulating the HPA and SAM axes. In this study, NYT treatment in NPY-KO zebrafish decreased the gene expression of *creb1* and CREB polypeptides but did not alter the phosphorylation of CREB. As the stimulation of glucocorticoid, DA, and NA receptors activate CREB (Xu et al., 2006; Wang et al., 2018), sustained decreases in these receptors by NYT might attenuate CREB polypeptide expression in NPY-KO zebrafish. In contrast, as CREB is known to positively regulate HPA and SAM-related genes (Paşca et al., 2011; Tafet and Nemeroff, 2020); suppression of the HPA/SAM axis could be induced by CREB downregulation.

The present study revealed that Cinnamon Bark and Polygala Root were responsible for the NYT-induced improvement of sociability in NPY-KO zebrafish. Their activities towards improvement in sociability were almost the same level. A previous study reported that Cinnamon Bark extract improves stress in a rat model of cold restraint stress (Saxena and Saxena, 2012) and despair behavior in depression model mice (Zada et al., 2016). Polygala Root improves memory impairment associated with scopolamine-induced amnesia in mice (Lee et al., 2015). However, there are few reports on improving social behavior, such as sociability with Cinnamon Bark and Polygala Root. Dysfunction of sociability is involved in disorders with high anxiety symptoms (Bowen et al., 2011). Our previous study found that NYT improved anxiety behavior in zebrafish. However, the effective herb in NYT was identified as Schisandra Fruit (Kawabe et al., 2021), different from this study. Previous study has shown that NPY-KO zebrafish do not exhibit anxious behavior under conditions of no external stress (Shiozaki et al., 2020). These studies indicate improvement of sociability in NYT treatment may be a mechanism distinct from the anxiolytic activity of NYT.

This study revealed that NYT improved sociability in zebrafish. However, several limitations should be noted. First, differences in drug digestion, absorption, and metabolism are not clearly defined between humans and fish. In zebrafish, gene expression profiles in the liver and gut microbiota are highly conserved with humans and mice (Hung et al., 2012). Glycosides are converted to aglycons in the zebrafish gut, followed by sulfation, glucuronidation, methylation, and other conjugations, similar to those in mammals (Hung et al., 2012). Several molecules involved in drug metabolism, such as CYPs and P-glycoprotein, are also conserved in zebrafish (Bresolin et al., 2005; Machado et al., 2014). However, studies on the kinetics of drugs after absorption and studies to identify enzymes involved in drug metabolism have rarely been conducted in zebrafish. In addition, zebrafish also possess unique isoforms of genes involved in drug metabolism (Tseng et al., 2005). Second, sociability-related proteins, such as OXT, VAP, GR, and TH1, are conserved in zebrafish, but the extent to which social regulatory systems are conserved between fish and mammals is controversial. Third, the structure of the zebrafish brain differs from that of humans, and the extent to which NYT is effective in the regulation of neural function is controversial. To address these limitations, it is highly desirable to identify the active components in Cinnamon Bark and Polygala Root, and their molecular actions on zebrafish neurons.

## 5 Conclusion

This study revealed that NYT treatment improved sociability in NPY-KO zebrafish, which may be due to the inactivation of the HPA and SAM axis. Furthermore, we found that Cinnamon Bark and Polygala Root improved sociability the most among the herbal medicines comprising NYT and thus may be a promising drug for treating sociability disorders.

## Data Availability

The raw data supporting the conclusion of this article will be made available by the authors, without undue reservation.

## Abbreviations

ACTH, adrenocorticotropic hormone; CREB, cAMP response element-binding protein; CRH, corticotropin-releasing hormone; DA, dopamine; GR, glucocorticoid receptor; HPA, hypothalamic-pituitary-adrenal; NA, noradrenaline; NYT, Ninjinyoeito; NPY, neuropeptide Y; POMC, pro-opiomelanocortin; SAM, sympathetic-adrenal-medullary.
