# Supplementary material for: Ninjinyoeito improves social behavior disorder in neuropeptide Y deficient zebrafish
Source: Front Pharmacol. 2022 Aug 12;13:905711. doi: 10.3389/fphar.2022.905711 (PMC9411948; doi:10.3389/fphar.2022.905711)
Supplement: Supplementary file 1 [file DataSheet1.pdf]

Supporting Information

Ninjinyoeito improves social behavior disorder in neuropeptide Y deficient zebrafish

Momoko Kawabe [a], Takumi Nishida [b], Chihoko Horita [b], Asami Ikeda [a], Ryuji Takahashi [c], Akio Inui [d] and Kazuhiro Shiozaki [a,b]

[a] Course of Biological Science and Technology, The United Graduate School of Agricultural Sciences, Kagoshima University, Kagoshima, Japan

[b] Department of Food Life Sciences, Faculty of Fisheries, Kagoshima University, Kagoshima, Japan

[c] Kampo Research Laboratories, Kracie Pharma, Ltd., Toyama, Japan

[d] Pharmacological Department of Herbal Medicine, Graduate School of Medical and Dental Sciences, Kagoshima University, Kagoshima, Japan

Supplementary Table 1 Primers used for real-time PCR

Supplementary Figure 1

3D-HPLC profile of NYT. Each chemical marker in the HPLC profile was identified by comparison with retention times and UV spectra (200–400 nm) of their reference standards.

Supplementary Figure 2

NPY-KO zebrafish were fed with control and herbal medicine-diet (equivalent to 3% NYT) twice a day for four days. Sociability was analyzed using the 3-chambers test. (A) Daily food intake. (B) Time spent in the empty chamber area. Control n = 41, herbal medicines n = 8. NPY-KO zebrafish were fed with control, Cinnamon Bark, and Polygala Root-diet (equivalent to 0.3% NYT) twice a day for four days. Sociability was analyzed using the 3-chambers test. (C) Daily food intake. (D) Total distance traveled. (E) Time spent in the empty chamber area. Control n = 8, herbal medicines n = 7. Results are shown as mean ± standard deviation. There is a significant difference between the different signs. n.s., not significant.

Supplementary Table 1 Primers used in this study

Gene	Primers	
th1	5′- TACATACGGCACGCTTCCTC - 3′	5′- GAACCGCACAGAAAACGGTC - 3′
th2	5′- AAAGGCTTATGGGGCTGGAC - 3′	5′- GCTGCAAGTGTAGGGGTCAT - 3′
orx	5′- TTCATGGCGCTGCTAGCTCA - 3′	5′- AATTTAGCGGGCTCCTCCAGC - 3′
cck	5′- AGCGTCAACCACCGGATAAA - 3′	5′- AGAGGACAGACGGAAACACG - 3′
crh	5′- TCGTCCATGATCTTGCGGTT - 3′	5′- GAATCTGCACGTGGTTGTCG - 3′
pomc	5′- CCCCCTACAAAATGACCCAT - 3′	5′- ATCCTTCCTCGGTTGGTCTT - 3′
gr	5′-AGCCGGAAAGGTAATCCAGC - 3′	5′-TCCAGCCCAGTCCAAAAGAC - 3′
avt	5′- CGCTCTCGTCTGCCTGCTAC - 3′	5′- TCTTAAGTCCCGCTGCTGCTG - 3′
ist	5′- TCTGGAAAGGCCTGCGGTTA - 3′	5′- GCTGTTGGCCGGTTGATTGA - 3
dat	5′- GCAGAACTTGTAGAAGGCGT- 3′	5′- TAGCTTCGCCACCTTCAATC- 3′
gad1b	5′- GCTACCAACCACAGGGAGAC- 3′	5′- CACAACGTCGAAACATAGCCG- 3′
gad2	5′- AACTTACCGCACCAAAACCT- 3′	5′-CCCACAACGACGCTACAATA - 3′
tph1a	5′-CGAGTAAAAGCGACGGGCCA - 3′	5′-TTCTTCGGGAACCACGGCAC - 3′
tph1b	5′- CTAAGAGCATACGGGGCTGG- 3′	5′- ACACCGAGGTGTGTGCTAAG- 3′
tph2	5′- CCTTGCATCCTCCATGCTCC- 3′	5′- CCCATCTGGCAATCCACCCA- 3′
bdnf	5′-TTCTGAGCACGGCAGAAGTT - 3′	5′-ACCTGTTGGAACATTTTCCCCTAT - 3′
creb1	5′- CGAGTCTGTGTCGGGCTATC- 3′	5′- CTTGTGCCAAGGTGGCAATC- 3′
actb	5′- CGCCATACAGACGAGAAGCCA - 3′	5′- AGCACCCTGTGCTGCTCACT - 3′

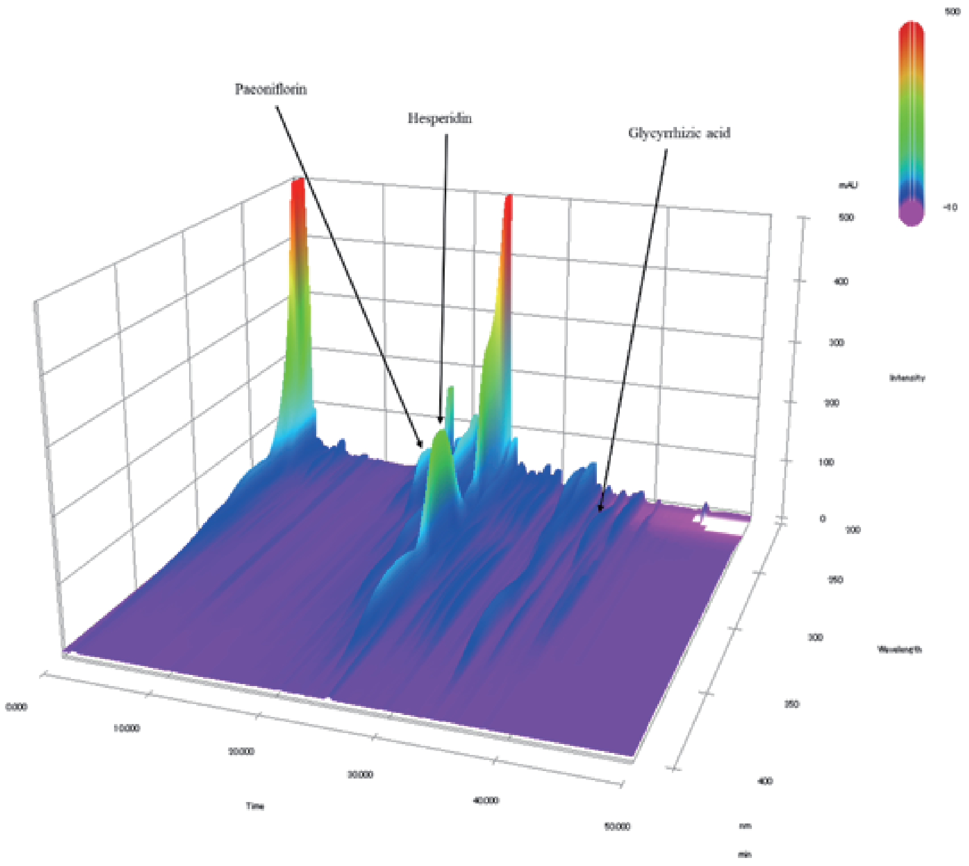

Supplemental fig. 1.

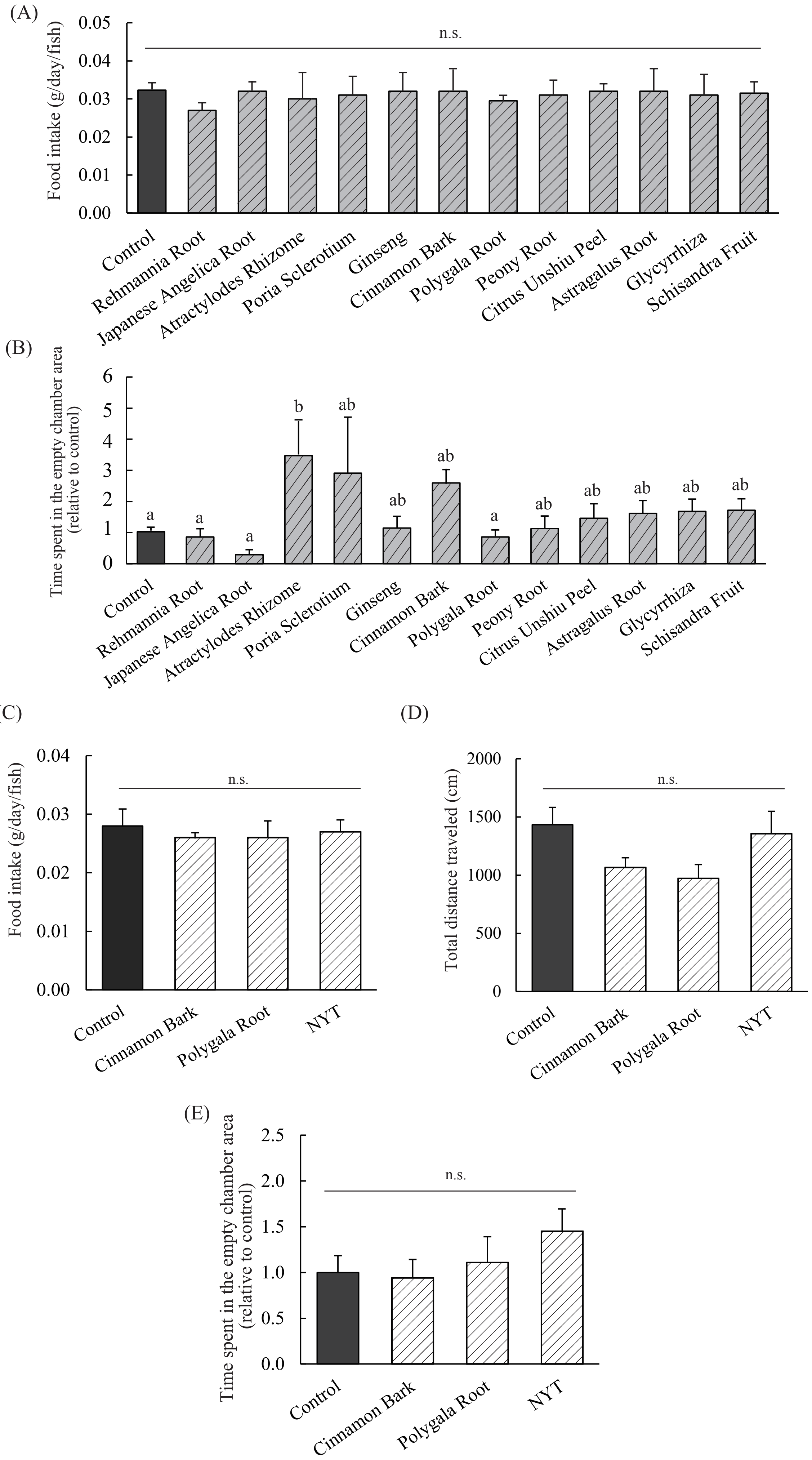

Supplemental fig. 2.
